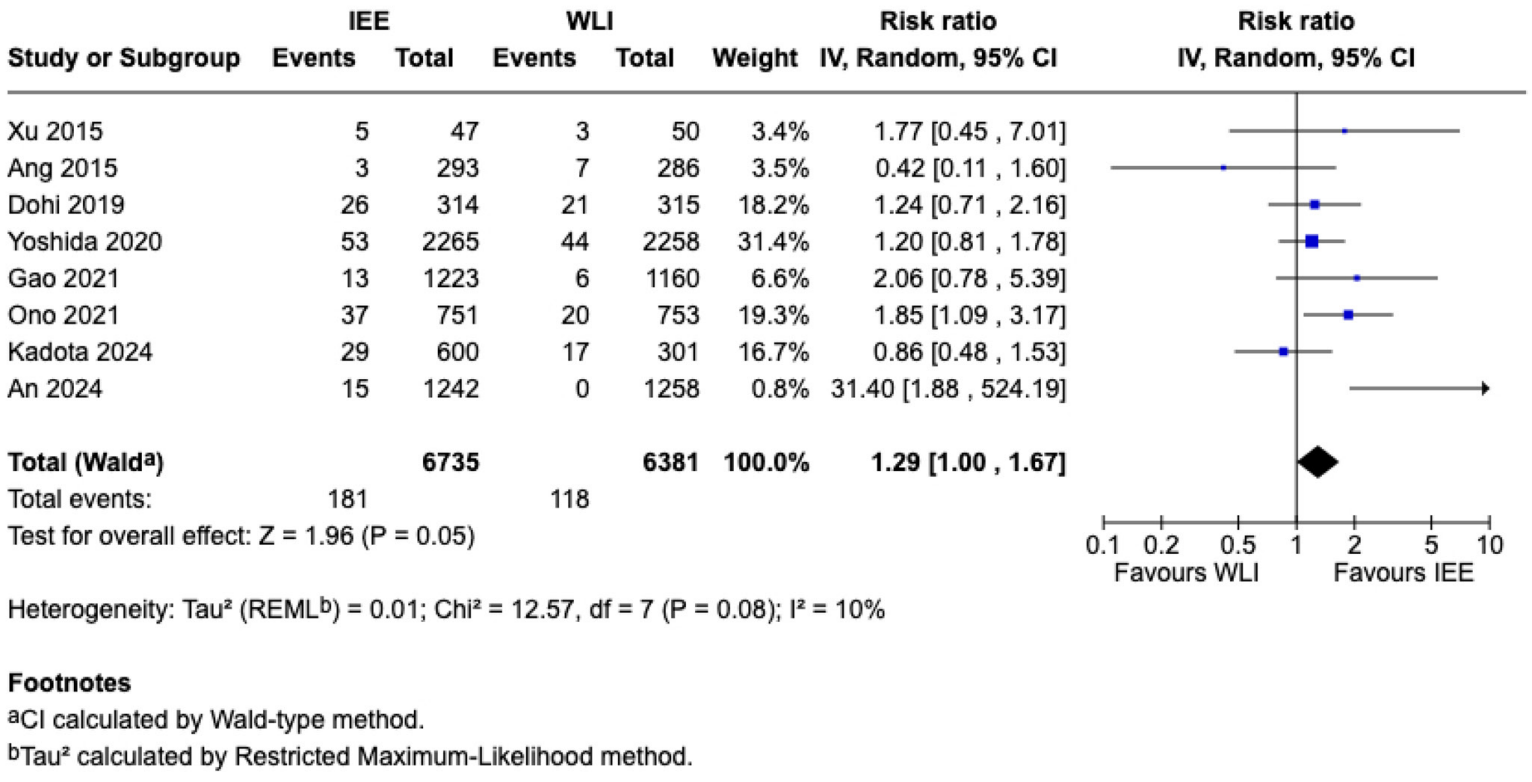# Poster Session I - A69 IMAGE-ENHANCED ENDOSCOPY VERSUS WHITE-LIGHT IMAGING FOR DETECTING EARLY GASTRIC CANCERS: A SYSTEMATIC REVIEW AND META-ANALYSIS

**DOI:** 10.1093/jcag/gwaf042.069

**Published:** 2026-02-13

**Authors:** J Watanabe, N Aumpan, Y Yuan, P Moayyedi

**Affiliations:** Department of Medicine, Division of Gastroenterology and Farncombe Family Digestive Health Research Institute, McMaster University, Hamilton, ON, Canada; Department of Medicine, Division of Gastroenterology and Farncombe Family Digestive Health Research Institute, McMaster University, Hamilton, ON, Canada; Department of Medicine, Division of Gastroenterology and Farncombe Family Digestive Health Research Institute, McMaster University, Hamilton, ON, Canada; Department of Medicine, Division of Gastroenterology and Farncombe Family Digestive Health Research Institute, McMaster University, Hamilton, ON, Canada

## Abstract

**Background:**

Imaged-enhanced endoscopy (IEE) improves mucosal and vascular visualization, making subtle neoplasia easier to see. However, the advantage of IEE over white-light imaging (WLI) in detecting early gastric cancer remains unclear.

**Aims:**

To assess the efficacy of IEE versus WLI for detecting early gastric cancer in patients who required esophagogastroduodenoscopy for screening, symptoms, or surveillance.

**Methods:**

We performed a random-effect meta-analysis of randomized controlled trials (RCTs) identified in MEDLINE, Embase, CENTRAL databases from inception to August 2025. Primary outcome was detection of early gastric cancer; secondary outcomes were detection of neoplastic lesions (cancer, high-grade intraepithelial neoplasia, and low-grade intraepithelial neoplasia) and *H. pylori* infection. Subgroup analysis of IEE type was performed. Risk of bias was assessed with RoB2; certainty of evidence was rated with GRADE. Protocol was registered (CRD420251131986).

**Results:**

We included 10 trials with 15,044 patients. IEE may slightly increase detection of early gastric cancer (8 RCT: relative risk [RR] 1.29, 95% confidence interval [CI] 1.00–1.67; number needed to screen [NNS] 187, 95% CI 81–∞; low-certainty evidence). IEE also may slightly increase neoplastic lesions (8 RCT: RR 1.47, 95% CI 1.15–1.87; NNS 65, 95% CI 35–202; low-certainty evidence). Evidence for effect of IEE on *H. pylori* detection is uncertain (one RCT: RR 1.19, 95% CI 1.00–1.41; NNS 7, 95% CI 4–∞; very low-certainty evidence). Subgroup results differed by modality; linked color imaging showed a favorable trend, but superiority remains unproven. Limitations include high risk of bias and imprecision because of the low event rate.

**Conclusions:**

IEE may slightly increase detection of early gastric cancer compared with WLI. Use of IEE can be considered in detecting early gastric cancer in high-risk populations.

**Funding Agencies:**

None